# Acute Type A Aortic Dissection with Concomitant Intracerebral Haemorrhage: A Therapeutic Dilemma Regarding the Timing of Surgery

**DOI:** 10.3390/jcdd13080371

**Published:** 2026-08-06

**Authors:** Yoganiranjana Dharuman, Borko Ivanov, Sami Sirat, Mirko Doss

**Affiliations:** 1 Department of Cardiothoracic Surgery, University Hospital Aachen, 52074 Aachen, Germany; 2 Department of Cardiothoracic Surgery, Helios Hospital Siegburg, 53721 Siegburg, Germany

**Keywords:** aortic aneurysm, aortic dissection, hemorrhagic stroke

## Abstract

Acute type-A aortic dissection is a life-threatening emergency requiring prompt surgical intervention. However, the management of patients presenting with concomitant intracerebral haemorrhage remains challenging, as the risk of neurological deterioration during surgery must be balanced against the risk of fatal aortic rupture. We report the rare case of a 68-year-old patient diagnosed with acute intracerebral haemorrhage involving the parietal lobe and right frontal region in association with acute type-A aortic dissection. Due to the neurological condition, a delayed surgical strategy was chosen. Six weeks after the initial diagnosis, supracoronary hemiarch replacement of the ascending aorta was successfully performed without postoperative neurological complications. This case highlights the complexity of decision-making in patients with simultaneous acute aortic pathology and intracranial bleeding. This educational report emphasises that immediate surgical repair remains the standard of care. The delayed strategy described here was dictated by an absolute contraindication to anticoagulation and reflects an exceptional, individualised management approach rather than a general therapeutic recommendation.

## 1. Introduction

The estimated incidence of aortic dissection is approximately 3–6 cases per 100,000 people per year, with an increasing prevalence in older populations. Aortic dissection occurs more frequently in men (65%) and has a mean age of presentation of approximately 63 years [1]. Acute aortic dissection and ruptured aortic aneurysm represent major causes of mortality among cardiovascular emergencies [2]. These conditions are classified as acute aortic syndromes, a group of life-threatening disorders involving the aorta, including aortic dissection, intramural haematoma without intimal tear, penetrating atherosclerotic ulcer, and impending or ruptured aortic aneurysm [3].

Risk factors for acute aortic syndromes are multifactorial and include hypertension, smoking, blunt chest trauma, genetic disorders associated with medial degeneration, drug abuse, inflammatory conditions, congenital abnormalities, and infectious diseases [4]. Intracerebral haemorrhage and acute type A aortic dissection share several risk factors, particularly hypertension and atherosclerosis. However, when acute aortic dissection is complicated by concomitant intracerebral haemorrhage, the optimal therapeutic strategy remains challenging. The risk of neurological deterioration due to cardiopulmonary bypass and systemic anticoagulation during surgery must be carefully balanced against the risk of aortic rupture. To date, the optimal timing of surgical intervention in this rare clinical scenario remains unclear [5].

## 2. Case Report

A 68-year-old woman with no family history of aortic disease was referred to our institution following an episode of chest pain and hemiparesis.

**Figure 1:**
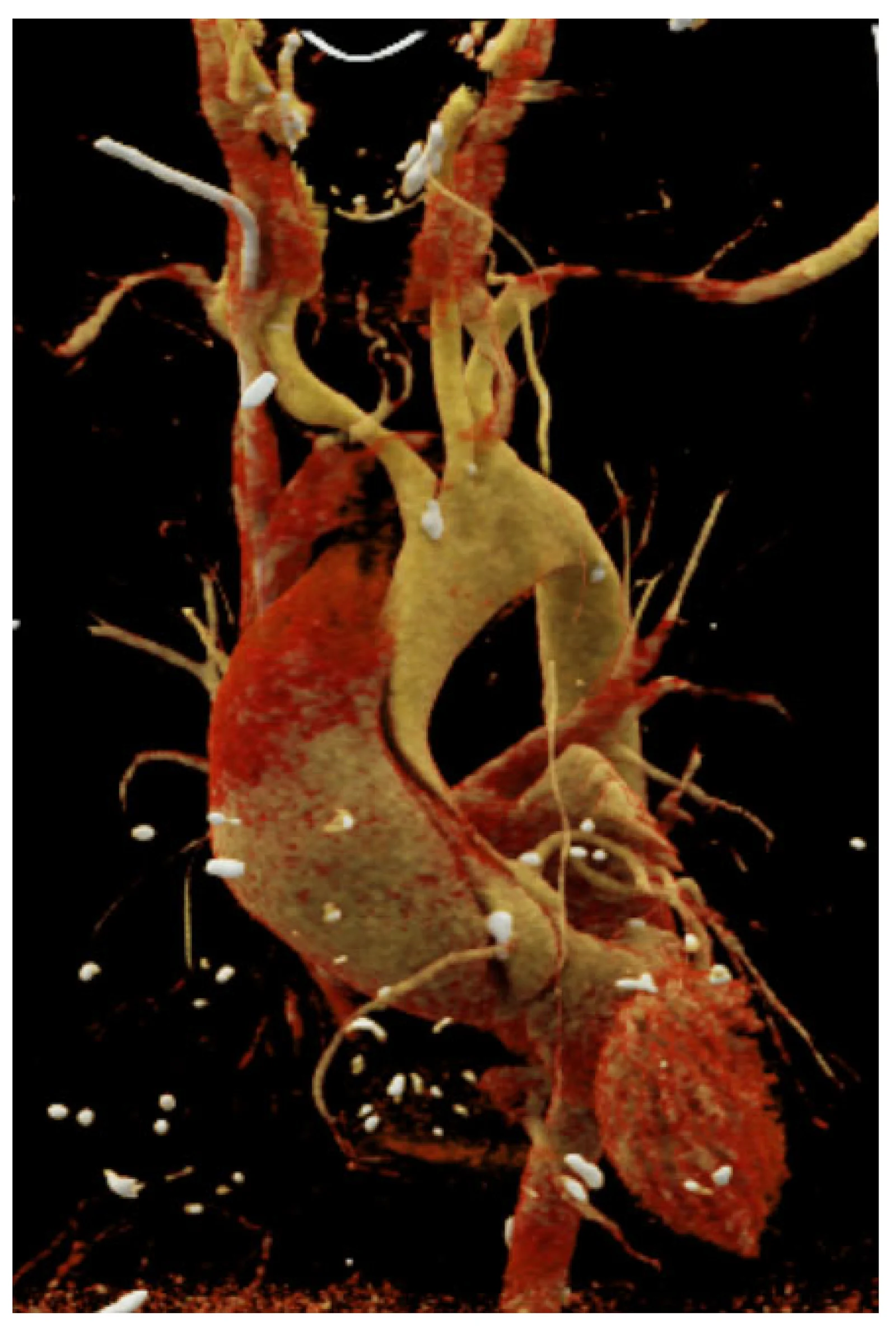
Reconstruction view of the Stanford type A aortic dissection. Three-dimensional reconstruction of computed tomography angiography demonstrating the acute Stanford type A aortic dissection.

Computed tomography (CT) imaging revealed a concomitant massive intracerebral haemorrhage involving the parietal and right frontal regions, together with an acute Stanford type A aortic dissection (Figure 1).

**Figure 2:**
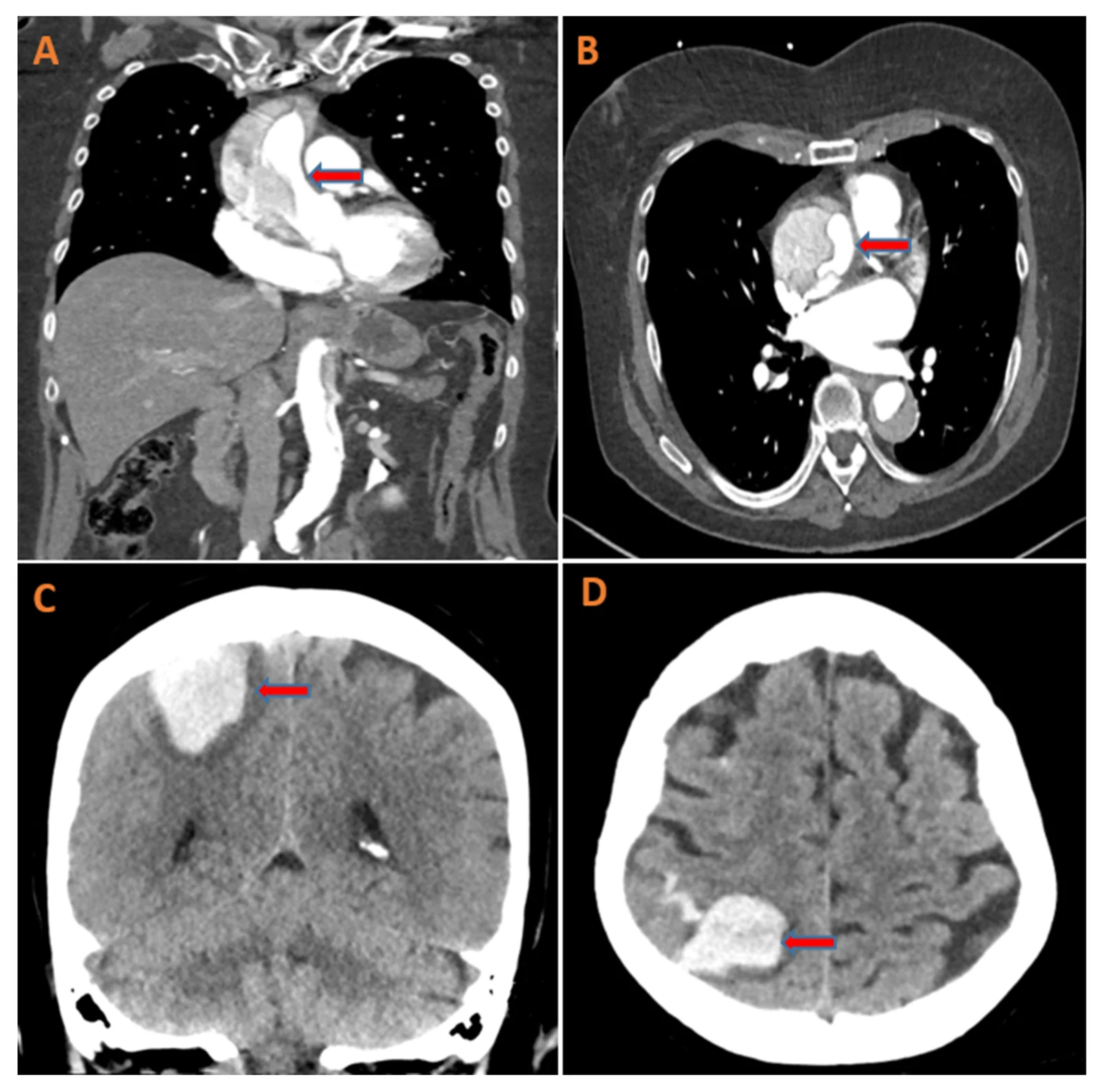
Preoperative computed tomography demonstrating Stanford type A aortic dissection (**A**,**B**) and intracerebral haemorrhage extending beyond the high parietal and right frontal regions (**C**,**D**).

Transthoracic echocardiography demonstrated aneurysmal dilatation of the ascending aorta with a clearly visible dissection flap. The left ventricle showed mild dilation with a left ventricular ejection fraction of 50% and no regional wall motion abnormalities. Assessment of valvular function revealed no aortic regurgitation. CT angiography confirmed a thoracic aortic aneurysm with acute type A dissection, a maximum diameter of 6.0 cm, significant displacement and compression of surrounding structures, and a large pericardial effusion with posterior displacement of the heart (Figure 2). Serial transthoracic echocardiographic examinations during intensive care monitoring were performed to assess cardiac function and haemodynamic stability; no relevant aortic valve insufficiency was detected.

Due to the concomitant intracranial haemorrhage and the risk of neurological deterioration associated with immediate surgical repair, a delayed surgical strategy was chosen. On admission, the patient was haemodynamically stable and required neither vasopressor nor inotropic support. The patient was admitted to the intensive care unit and underwent close neurological and cardiovascular monitoring. Serial CT scans were initially performed twice weekly and subsequently once weekly to assess the progression of intracerebral haemorrhage. Blood pressure was controlled using a continuous urapidil infusion, targeting a systolic pressure below 120 mmHg in accordance with anti-impulse therapy for acute aortic dissection while maintaining adequate cerebral and systemic perfusion. Heart rate control was not required, as the patient remained in normofrequent sinus rhythm throughout the observation period. These targets were chosen to balance cerebral and end-organ (e.g., renal) perfusion against the risk of dissection progression and re-bleeding. During the first three weeks, therapeutic anticoagulation was withheld because of the intracranial haemorrhage. During this period, venous thromboembolism prophylaxis consisted exclusively of graduated compression stockings. After radiological confirmation of haemorrhage stability, prophylactic-dose subcutaneous unfractionated heparin was initiated for venous thromboembolism prophylaxis. The treatment strategy was established following interdisciplinary discussion involving cardiac surgery, neurosurgery, neurology, anaesthesia, and intensive care specialists.

**Figure 3:**
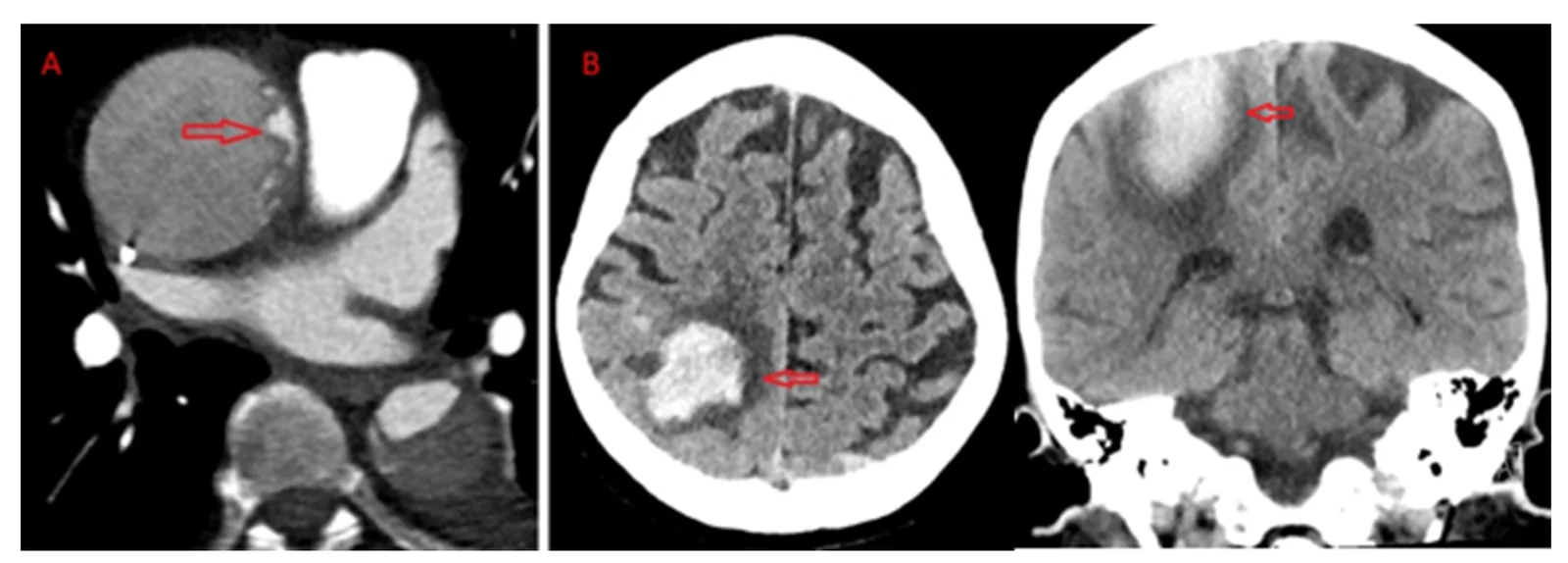
CT imaging two weeks after admission showing stable Stanford type A dissection (**A**) with enlargement of both cerebral haemorrhages (**B**).

During the second week of observation, follow-up CT angiography demonstrated progressive compression of the true lumen by the false lumen, although the anatomical extent of the dissection remained unchanged (Figure 3).

**Figure 4:**
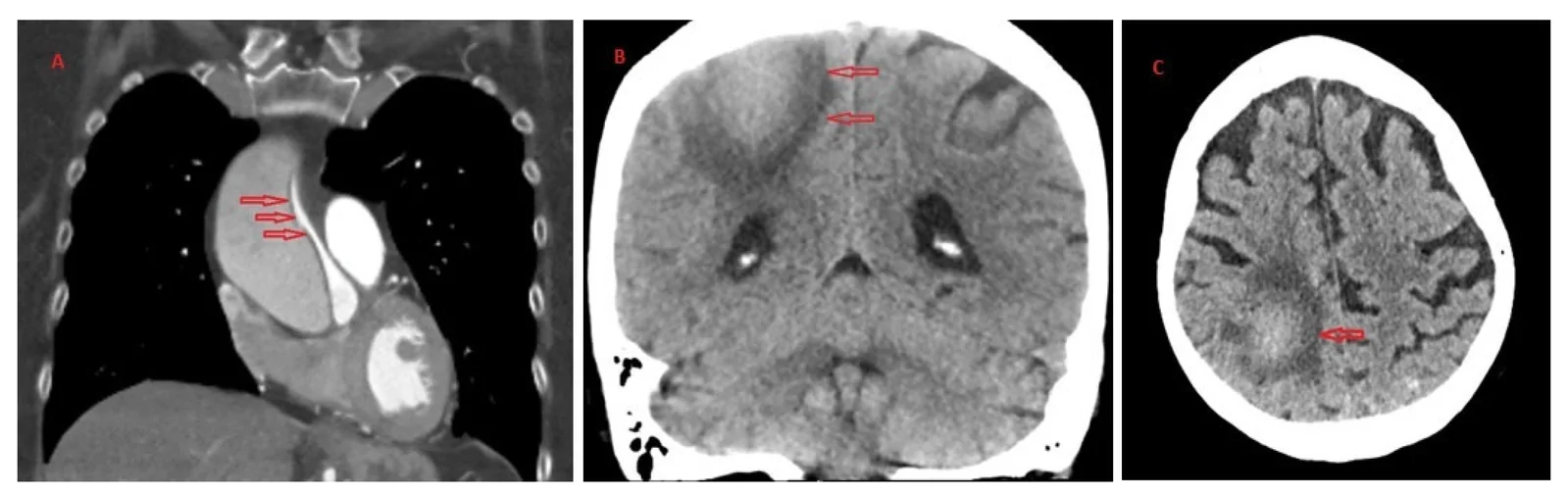
CT imaging four weeks after admission showing progressive collapse of the true lumen (**A**) and beginning resorption of the intracerebral haematoma (**B**,**C**).

After four weeks, follow-up imaging demonstrated gradual resorption of the intracerebral haematoma. However, progressive compression of the true aortic lumen was observed (Figure 4).

**Figure 5:**
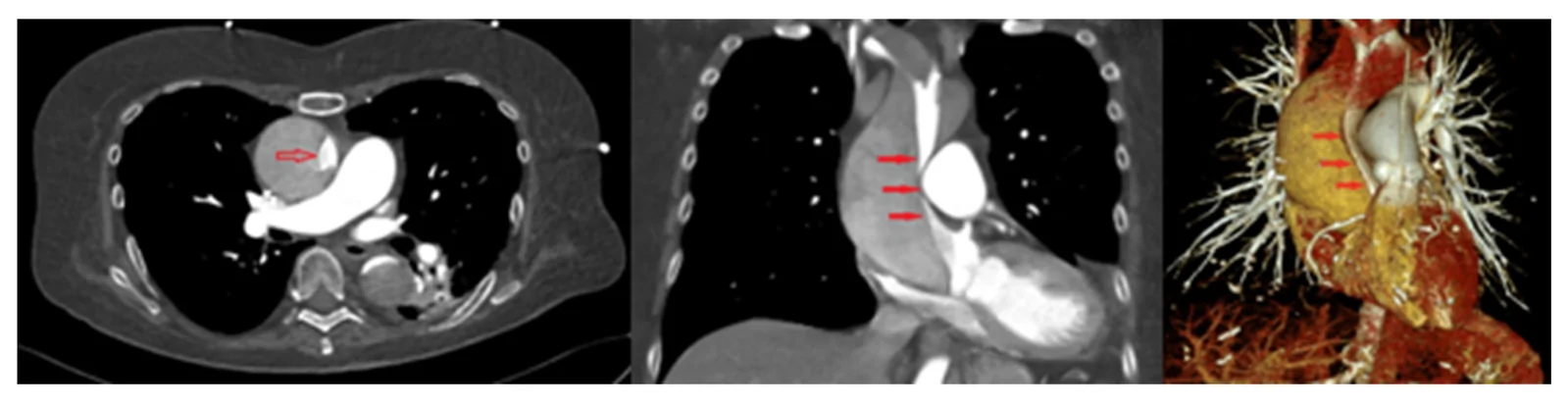
CT imaging after six weeks demonstrating the remaining true lumen of the aorta.

The findings were repeatedly discussed within the multidisciplinary team, including neuroradiology, until regression of the haemorrhage and absence of ongoing bleeding activity were confirmed. Because cardiopulmonary bypass mandates full heparinisation, which is absolutely contraindicated in the presence of an acute intracerebral haemorrhage, surgery was deferred until the haemorrhage was radiologically stable and the neurosurgical team explicitly cleared the patient for surgery under full anticoagulation. The earliest possible time point after this clearance was chosen; earlier intervention would have been undertaken only as a bail-out in the event of imminent aortic rupture. After six weeks, the patient was considered suitable for surgical intervention, and the procedure was scheduled for the following day (Figure 5).

**Figure 6:**
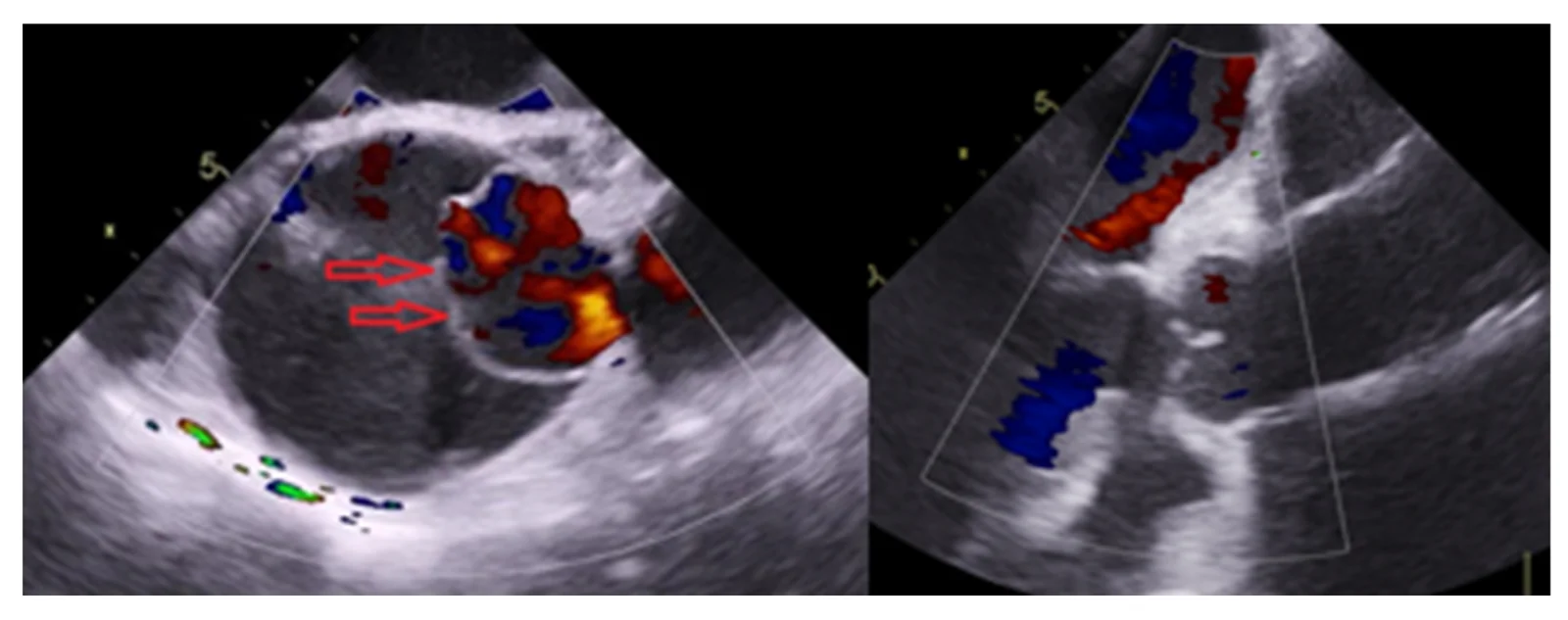
Intraoperative TEE demonstrating the dissection membrane and postoperative findings following supracoronary hemiarch replacement.

Intraoperatively, thrombotic material within the pericardium and a large ascending aortic aneurysm occupying the majority of the pericardial space with posterior displacement of the heart were identified. Supracoronary replacement of the ascending aorta with hemiarch replacement was performed under cardiopulmonary bypass with near-infrared spectroscopy (NIRS) monitoring. Arterial and venous cannulation were both established via the right femoral vessels. Myocardial protection was achieved using retrograde administration of Bretschneider (HTK) cardioplegic solution via the coronary sinus, followed by selective antegrade administration into the left and right coronary ostia. Cardiopulmonary bypass time was 191 min, aortic cross-clamp time was 85 min, and total circulatory arrest with selective antegrade cerebral perfusion was maintained for 32 min at a core temperature of 28 °C. Systemic anticoagulation during cardiopulmonary bypass was achieved with unfractionated heparin under activated clotting time (ACT) monitoring and was reversed with protamine after completion of the procedure. NIRS values remained stable throughout the procedure. Intraoperative transoesophageal echocardiography (TEE) confirmed preserved aortic valve competence (Figure 6). A supracoronary ascending aortic replacement with hemiarch replacement was chosen because the aortic root was not dilated, and the native aortic valve was structurally normal without calcification or insufficiency. Furthermore, the distal aortic arch was of normal calibre without relevant pathological involvement. Therefore, more extensive arch replacement, including a frozen elephant trunk procedure, was not indicated.

**Figure 7:**
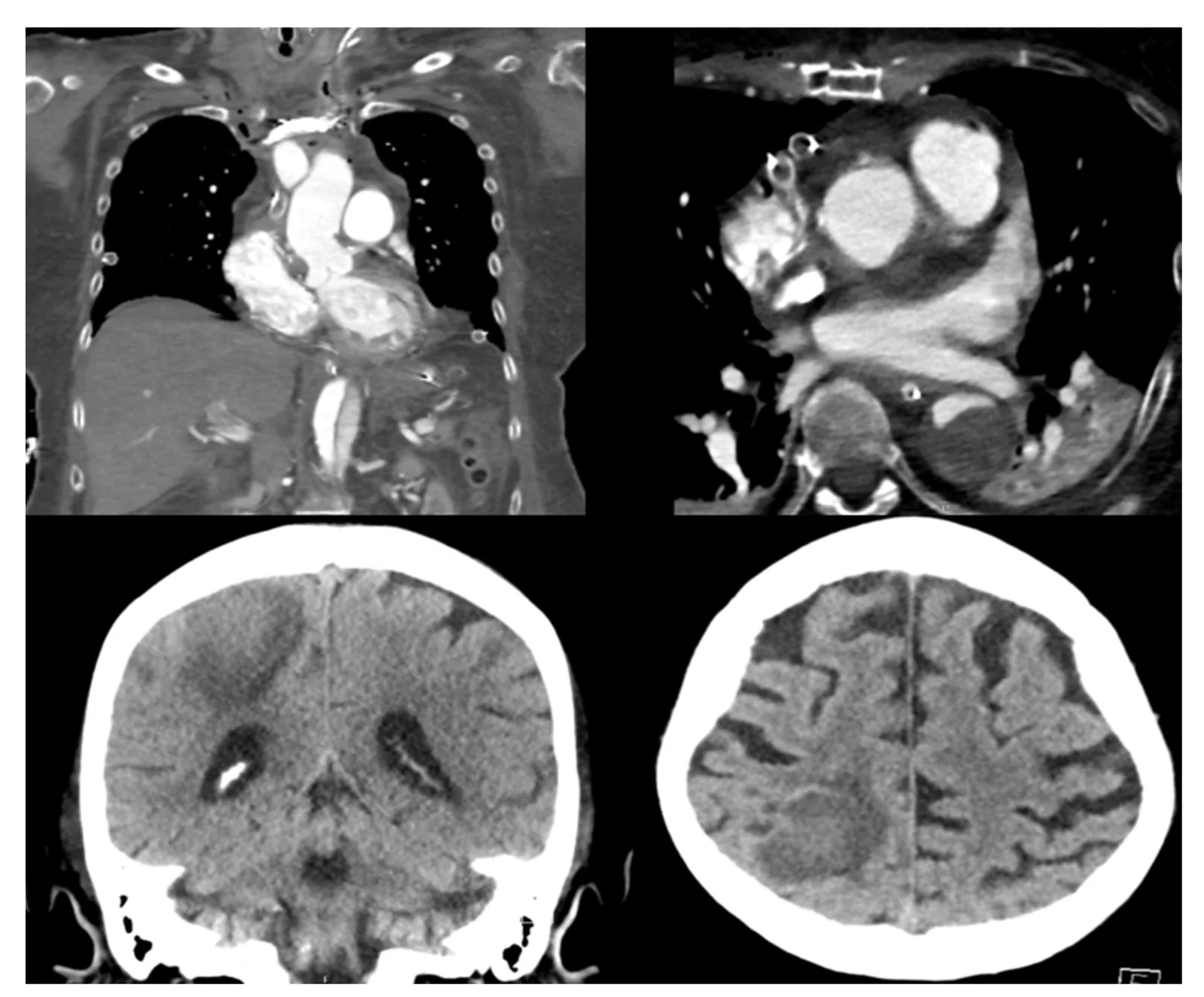
Postoperative computed tomography demonstrating regression of the cerebral haemorrhage and correct positioning of the prosthesis after supracoronary hemiarch replacement of the ascending aorta.

The patient remained haemodynamically stable during the procedure and was transferred to the intensive care unit. Following successful supracoronary ascending aortic and hemiarch replacement, heparin-induced thrombocytopenia (HIT) was diagnosed. Anticoagulation was therefore performed using a continuous argatroban infusion. In view of the recent intracranial haemorrhage, only prophylactic anticoagulation was targeted, with the argatroban dose adjusted to achieve a prophylactic rather than a therapeutic activated partial thromboplastin time (aPTT). The postoperative course was otherwise uncomplicated. The patient was discharged on postoperative day 7 following postoperative CT imaging, which demonstrated regression of the intracerebral haemorrhage and correct positioning of the vascular prosthesis (Figure 7). No neurological deficits were observed. Follow-up was limited to the in-hospital course. At discharge, the patient remained free of neurological deficits, and no recurrent intracranial haemorrhage was observed.

This case report was prepared in accordance with the CARE (CAse REport) guidelines [6] (see Supplementary Materials, Checklist S1).

## 3. Discussion

The management of ascending aortic aneurysms and acute type A aortic dissection remains a major challenge in cardiovascular surgery and has historically been associated with considerable mortality. The development of advanced surgical techniques, including improved graft materials, optimized myocardial protection strategies, retrograde cerebral perfusion during circulatory arrest, intraoperative transoesophageal echocardiography, and improved control of haematological factors, has contributed to a significant reduction in perioperative mortality.

Immediate surgical repair remains the standard of care for patients presenting with acute type A aortic dissection. However, the presence of a simultaneous intracerebral haemorrhage creates a unique therapeutic dilemma. The need for cardiopulmonary bypass and systemic anticoagulation during surgery may increase the risk of neurological deterioration, while delaying surgery carries the potential risk of aortic rupture or progression of the dissection. Therefore, in selected cases such as the present one, the individual risk assessment may favour a delayed surgical strategy under strict clinical and radiological surveillance.

The 2024 EACTS/STS Guidelines for the aortic organ [7] assign a Class I recommendation to emergency surgery for acute type A aortic dissection and a Class IIa recommendation to open repair even in the presence of neurological deficits. These recommendations, however, pertain to ischaemic neurological deficits and malperfusion, whereas our patient presented with an intracerebral haemorrhage, which represents an absolute contraindication to the full heparinisation required for cardiopulmonary bypass. No current guideline specifically addresses the combination of acute type A dissection and concurrent intracerebral haemorrhage. From the multidisciplinary perspective, immediate surgery offers protection against aortic rupture but carries a high risk of catastrophic re-bleeding and neurological deterioration under anticoagulation, whereas a delayed strategy mitigates the neurological risk at the cost of ongoing exposure to aortic rupture and true-lumen collapse. In our patient, this balance favoured a monitored delay until neurosurgical clearance. Although direct evidence for anticoagulation management in this specific setting is lacking, data from other high-risk indications, such as mechanical heart valves, suggest that resumption of anticoagulation after intracranial haemorrhage may be feasible in selected patients [8]. In our patient, the six-week interval reflected a conservative approach given the competing risks.

This rare case highlights the importance of a patient-specific approach involving timely diagnosis, careful preoperative management of associated complications, appropriate surgical planning, and meticulous peri- and postoperative care. Both intracerebral haemorrhage and acute aortic dissection share several risk factors, including hypertension and atherosclerosis; however, the simultaneous occurrence of both conditions remains uncommon and represents a significant therapeutic challenge. In our patient, the decision to postpone surgical intervention was based on multidisciplinary evaluation, continuous monitoring, and confirmation of haemorrhage stabilization, ultimately allowing successful repair without neurological complications.

The optimal timing of surgery in patients presenting with acute type A aortic dissection complicated by intracerebral haemorrhage remains uncertain. Due to the rarity of this clinical scenario, management decisions should be made on an individual basis through close collaboration between cardiovascular surgeons, neurologists, neurosurgeons, and anaesthesiologists, carefully balancing the risks of immediate intervention against those of delayed repair.

### Limitations

This is a single educational case report and cannot establish general management recommendations; negative outcomes of similar strategies may be under-reported. The delayed approach described here should not be generalised, as immediate surgery remains the standard of care for acute type A aortic dissection. Follow-up was limited to the in-hospital course, and longer-term outcomes are not available.

## 4. Conclusions

Acute type A aortic dissection complicated by intracerebral haemorrhage represents an exceptionally challenging clinical scenario without established evidence-based management pathways. Treatment decisions should be individualized through close multidisciplinary collaboration, balancing the immediate risk of aortic rupture against the potential for neurological deterioration associated with systemic anticoagulation. This case demonstrates that, in highly selected patients with stable neurological findings and intensive clinical and radiological surveillance, delayed surgical repair may be considered when immediate intervention is deemed prohibitively hazardous. However, immediate surgical repair remains the standard of care for acute type A aortic dissection, and the delayed strategy described here should be regarded as an exceptional, patient-specific approach rather than a general treatment recommendation.

## Data Availability

The data presented in this study are available on request from the corresponding author.

## Supplementary Materials

The following supporting information can be downloaded at: https://www.mdpi.com/article/10.3390/jcdd13080371/s1, CARE Checklist, Supplementary Material—Acute type A aortic dissection with concomitant intracerebral haemorrhage.
