# Supplementary material for: Acute Type A Aortic Dissection with Concomitant Intracerebral Haemorrhage: A Therapeutic Dilemma Regarding the Timing of Surgery
Source: J Cardiovasc Dev Dis. 2026 Aug 6;13(8):371. doi: 10.3390/jcdd13080371 (PMC13513898; doi:10.3390/jcdd13080371)
Supplement: Supplementary file 1 [file jcdd-13-00371-s001.zip › jcdd-4440596-supplementary.pdf]

# CARE Checklist, Supplementary Material

*Acute type A aortic dissection with concomitant intracerebral haemorrhage*

| **Topic** | **Item** | **Checklist item description** | **Reported (section)** |
|---|---|---|---|
| Title | 1 | The words "case report" appear in the title, with the area of focus. | Title |
| Key words | 2 | 2–5 key words identifying the diagnoses/interventions. | Key words |
| Abstract | 3a | Introduction — what is unique and what it adds. | Abstract |
| | 3b | Main symptoms/clinical findings, diagnoses, interventions, outcomes. | Abstract |
| | 3c | Conclusion — main "take-away" lessons. | Abstract |
| Introduction | 4 | Brief background summary referencing the medical literature. | Introduction |
| Patient information | 5a | De-identified demographic and other patient information. | Case report |
| | 5b | Main concerns and symptoms of the patient. | Case report |
| | 5c | Medical, family and psychosocial history including relevant genetic information. | Case report |
| | 5d | Relevant past interventions and their outcomes. | Case report |
| Clinical findings | 6 | Relevant physical examination and clinical findings. | Case report |
| Timeline | 7 | Important dates/times in the episode of care (figure/table). | Case report / Figures 1–7 |
| Diagnostic assessment | 8a | Diagnostic methods (physical exam, laboratory, imaging, questionnaires). | Case report / Figures |
| | 8b | Diagnostic challenges. | Case report / Discussion |

| | | | |
|---|---|---|---|
| | 8c | Diagnostic reasoning including other diagnoses considered. | Case report / Discussion |
| | 8d | Prognostic characteristics where applicable. | Case report / Discussion |
| Therapeutic intervention | 9a | Types of intervention (pharmacologic, surgical, preventive, self-care). | Case report |
| | 9b | Administration of intervention (dosage, strength, duration). | Case report |
| | 9c | Changes in intervention with explanations. | Case report |
| Follow-up and outcomes | 10a | Clinician- and patient-assessed outcomes. | Case report |
| | 10b | Important follow-up diagnostic and other test results. | Case report / Figure 7 |
| | 10c | Intervention adherence and tolerability (how assessed). | Case report |
| | 10d | Adverse and unanticipated events. | Case report |
| Discussion | 11a | Strengths and limitations of the management of this case. | Discussion / Limitations |
| | 11b | Relevant medical literature. | Discussion |
| | 11c | The rationale for conclusions. | Discussion / Conclusion |
| | 11d | The main “take-away” lessons of this case report. | Conclusion |
| Patient perspective | 12 | The patient should share their perspective on their care (where appropriate). | Not applicable |
| Informed consent | 13 | Did the patient give informed consent? Provide if requested. | Informed Consent Statement |

*Reference: Gagnier JJ, Kienle G, Altman DG, Moher D, Sox H, Riley D; CARE Group. The CARE guidelines: consensus-based clinical case reporting guideline development. BMJ Case Rep. 2013. (6)*
